# A Mutation in Intracellular Loop 4 Affects the Drug-Efflux Activity of the Yeast Multidrug Resistance ABC Transporter Pdr5p

**DOI:** 10.1371/journal.pone.0029520

**Published:** 2012-01-06

**Authors:** Xiaoxian Guo, Jingkai Li, Tanjun Wang, Zhenhua Liu, Xin Chen, Yudong Li, Zhenglong Gu, Xuming Mao, Wenjun Guan, Yongquan Li

**Affiliations:** 1 College of Life Sciences, Zhejiang University, Hangzhou, People's Republic of China; 2 Division of Nutritional Sciences, Cornell University, Ithaca, New York, United States of America; 3 College of Food Science and Biotechnology, Zhejiang Gongshang University, Hangzhou, People's Republic of China; Technion-Israel Institute of Technology Haifa 32000 Israel, Israel

## Abstract

Multidrug resistance protein Pdr5p is a yeast ATP-binding cassette (ABC) transporter in the plasma membrane. It confers multidrug resistance by active efflux of intracellular drugs. However, the highly polymorphic Pdr5p from clinical strain YJM789 loses its ability to expel azole and cyclohexmide. To investigate the role of amino acid changes in this functional change, *PDR5* chimeras were constructed by segmental replacement of homologous BY4741 *PDR5* fragments. Functions of *PDR5* chimeras were evaluated by fluconazole and cycloheximide resistance assays. Their expression, ATPase activity, and efflux efficiency for other substrates were also analyzed. Using multiple lines of evidence, we show that an alanine-to-methionine mutation at position 1352 located in the predicted short intracellular loop 4 significantly contributes to the observed transport deficiency. The degree of impairment is likely correlated to the size of the mutant residue.

## Introduction

ATP-binding cassette (ABC) transporters are integral membrane proteins that carry diverse substrates across plasma membrane [1], [2]. Active extrusion of xenobiotics mediated by ABC transporters is one of the most common resistance mechanisms developed by microorganisms. [3], [4], [5]. As the most abundant ABC transporter in *Saccharomyces cerevisiae*, Pdr5p plays a major role in cellular detoxification via efflux of a wide variety of drugs and substrates in an ATP-dependent manner [6], [7], [8], [9]. Predicted topology of Pdr5p shows that it carries two highly hydrophobic transmembrane domains (TMDs) and two nucleotide binding domains (NBDs) [10], [11]. In a functional Pdr5p transporter, several amino acid residues of the TMDs determine substrate specificity [12], whereas some other amino acid residues of the NBDs are responsible for nucleotide binding and/or hydrolysis, then couple the liberated energy to conformational changes at the TMDs, which allow the translocation of drug substrates across plasma membrane barrier [13], [14]. Residues in NBDs may also selectively affect substrate selection [15]. The mechanism for how Pdr5p recognizes and transports a large number of structurally distinct substrates still remains unclear [16].

Previous reports have described many site-directed mutations in NBDs or TMDs of Pdr5p [11], [12], [13], [15], [17], [18], [19], leading to elucidation of the roles of single amino acid in the overall function of the protein. These studies emphasized on alterations affecting ATP hydrolysis and/or binding, as well as transport of substrates through the membrane. Pathogenic strain YJM789 was derived from a clinical *S. cerevisiae* isolate (YJM128) collected from the lung of an AIDS patient [20]. *PDR5* in YJM789 strain contains 274 nucleotide differences (5.3% divergence) resulting in 80 amino acid changes from *PDR5* in the lab strain BY4741 (hereafter referred to as *BPDR5*), whereas at the whole genome level the nucleotide difference between these two strains is only 0.43% [21]. Interestingly, most of the amino acid differences occur in two TMDs [22]. Our earlier work presented evidence that the drug-efflux function of Pdr5p in YJM789 strain is impaired by its dramatic sequence divergence although YJM789 Pdr5p localized to plasma membrane similarly to BY4741 Pdr5p. Further study illustrated that the sequence divergence in the TMD2 segment leads to fluconazole and cycloheximide hypersensitivity of YJM789, which contains a part of the predicted C-terminal NBD and the whole C- terminal TMD coding regions of *PDR5* in YJM789 strain (hereafter referred to as *YPDR5*) [22].

In this study, the mutated sites in *YPDR5* TMD2 segment that impair Pdr5p drug-efflux function were traced by the segment replacement method. The results show an A1352M mutation in the predicted intracellular loop 4 (ICL4) [11], [23], [24], [25] can cause severe decrease of drug export ability.

## Results

### Mutation sites in block S2A and S2B are critical for Pdr5p-mediated drug resistance

**Figure 1:**
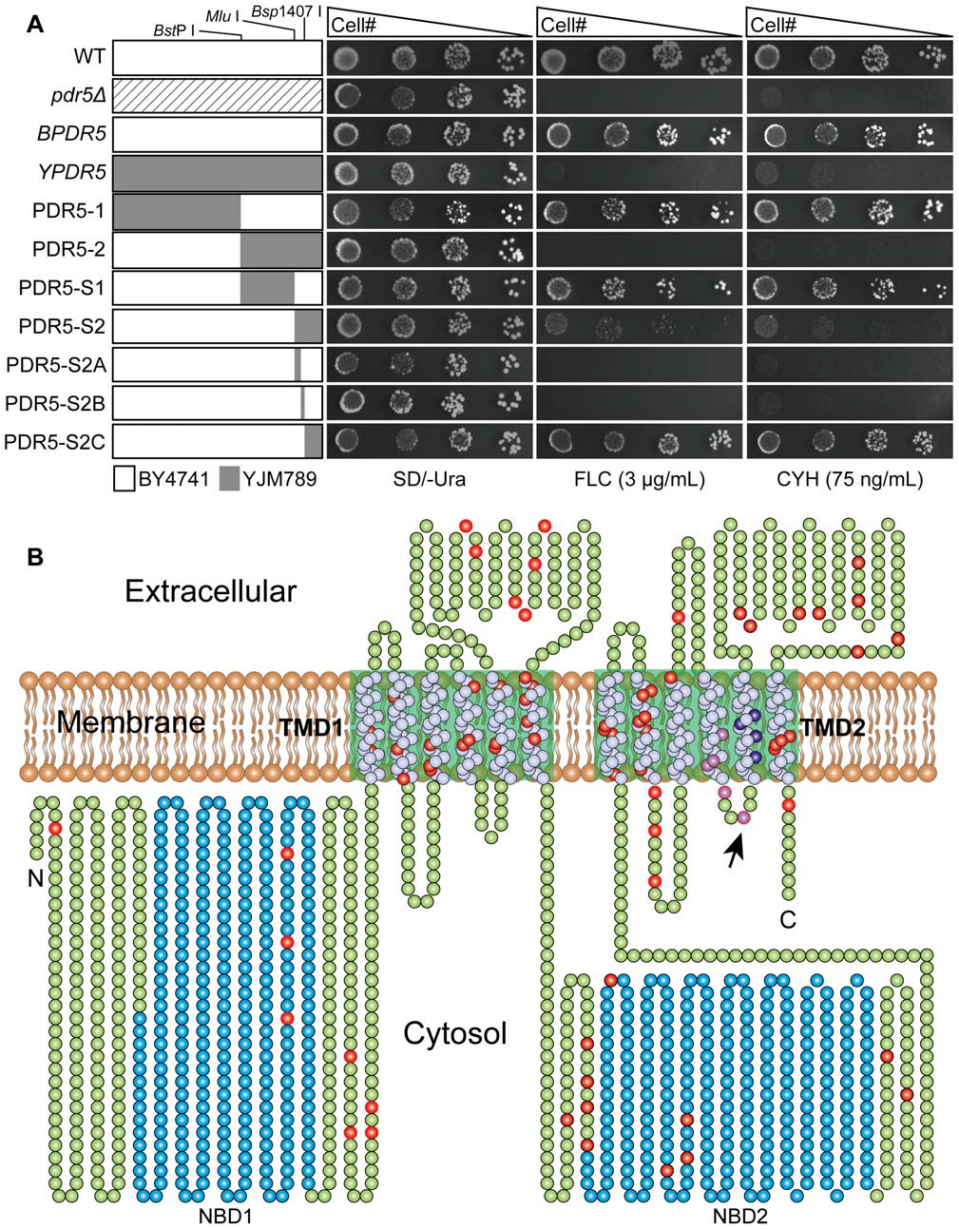
Identification of residues important for Pdr5p function and distribution of mutations in YJM789 Pdr5p. (A) Segmental replacement of BY4741 *PDR5* and drug resistance profiles of *pdr5Δ S. cerevisiae* cells expressing wild type and chimeric *PDR5s* by spot assay. Various regions of BY4741 *PDR5* are segmentally replaced by corresponding segments from *YPDR5*. Segments from BY4741 and YJM789 *PDR5* are shown as white and grey rectangles, respectively. The unique *Bsp* I, *Mlu* I, *Bsp1407* I restriction sites, which were used to construct *PDR5* chimeras are marked. Five-fold serial dilutions of *pdr5Δ* mutant carrying different *PDR5* constructs were spotted on SD/-Ura media in the absence and the presence of 3 µg/mL fluconazole or 75 ng/mL cycloheximide. Plates were incubated for 72 hours at 30°C. (B) The topological model is based on the molecular modeling study of Pdr5p [11]. Two-dimensional schematic cartoon of Pdr5p shows the 12 predicted membrane spanning helices (white spheres) and two NBDs (blue spheres). The green boxed areas indicate the TMDs. Divergent residues between BY4741 and YJM789 are shown as red, purple and dark blue spheres, respectively. Ala 1352 is indicated by black arrow.

Previous studies found that Pdr5p in YJM789 strain cannot export its drug substrates such as cyclohexmide and fluconazole efficiently [22], [26]. In this study, *YPDR5* and *BPDR5* were overexpressed from high-copy plasmids. As shown in Fig. 1A, cells expressing *BPDR5* were able to grow as well as wild type BY4741 on media containing 3 µg/mL fluconazole or 75 ng/mL cyclohexmide, while cells expressing *YPDR5* were phenotypically similar to the *pdr5* null mutant G1. It shows that the dramatic sequence divergence in *YPDR5* disrupts its drug-efflux function under overexpression background. By exchanging of NBD-TMD coding regions in *BPDR5* with their counterparts in *YPDR5*, *PDR5* chimeras *PDR5-1* and *PDR5-2* were constructed. Drug susceptibility analysis revealed that cells expressing *PDR5-1* had similar drug resistance patterns as cells expressing *BPDR5*, while cells expressing *PDR5-2* was as same as *pdr5* null mutant. This result suggests that sequence divergence in the C-terminal NBD-TMD region of *YPDR5* can abrogate the Pdr5p-mediated drug resistance.

To identify which amino acid residues are important for the efflux function of Pdr5p, additional *PDR5* chimeras *PDR5-S1* and *PDR5-S2* were created by dividing the C-terminal half divergent sequence of *PDR5-2* into two segments S1 and S2. After transforming *PDR5-S1* or *PDR5-S2* overexpression plasmid into the fluconazole-sensitive *pdr5* null mutant G1, their drug sensitivity to fluconazole was tested. As shown in Fig. 1A, cells expressing *PDR5-S1* presented the similar drug resistant phenotypes as cells expressing *BPDR5*, whereas cells expressing *PDR5-S2* were highly susceptible to fluconazole. Hence, sequence divergence in the segment S2 from *YPDR5* can cause reduction in Pdr5p-mediated fluconazole resistance.

In order to narrow down the critical regions for Pdr5p function, segment S2 from *YPDR5* was subdivided into 3 blocks (S2A, S2B and S2C) to replace their homologous regions in *BPDR5* and the resulted *PDR5* chimeras were designated as *PDR5-S2A*, *PDR5-S2B* and *PDR5-S2C*, respectively. Comparison of drug resistance phenotypes shows that the replacement of block S2C did not affect the Pdr5p-mediated fluconazole efflux, while *PDR5-S2A* and *PDR5-S2B* resulted in hypersusceptibility to fluconazole and cyclohexmide as compared to *BPRD5* (Fig. 1A). Sequence analysis shows that *PDR5-S2A* and *PDR5-S2B* possess 5 (Fig. 1B, purple spheres) and 4 amino acid mutations (Fig. 1B, dark blue spheres), respectively. These changed residues could disturb Pdr5p–mediated drug efflux.

### A1352M mutant strain is sensitive to fluconazole and cyclohexmide

**Figure 2:**
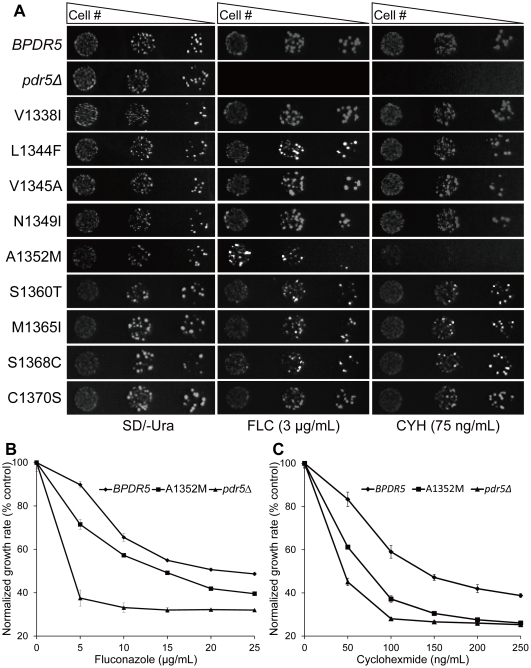
Drug resistance profiles of cells expressing *PDR5* variants. (A) Nine mutated sites distributed throughout blocks S2A and S2B were separated by site-directed mutagenesis and fluconazole sensitivities of mutant variants were determined by spot assay. Cells were grown overnight and reinoculated to OD600 = 0.1, then 4 µL of five-fold serial dilutions were spotted onto drug free or drug containing (3 µg/mL fluconazole or 75 ng/mL cycloheximide) SD/-Ura plates. The plates were incubated at 30°C for 72 hours. (B) Resistance of *BPDR5* (⧫), *pdr5Δ* (▴) and A1352 (▪) mutants to increasing concentrations of fluconazole. Cells from overnight cultures were inoculated to OD600 = 0.1. Growth was estimated on the basis of OD600 and plotted as percentage growth, where the growth in drug free media was taken to be 100%. The mean values of three independent experiments are plotted and the error bars represent the S.D. (C) Growth of *BPDR5* (⧫), *pdr5Δ* (▴) and A1352 (▪) mutants was compared as described in B, except that the experiment was performed in the presence of increasing concentrations of cycloheximide.

In order to investigate which mutations in the S2A and S2B regions can alter the capacity of Pdr5p to confer drug resistance, as well as whether there are any additive or synergistic effects in mutation combinations, nine mutants expressing variants of *PDR5* including V1338I, L1344F, V1345A, N1349I, A1352M, S1360T, M1365I, S1368C and C1370S were constructed (See Table 1). The drug resistance patterns of all above Pdr5p mutants were indistinguishable from WT except A1352M mutant (Fig. 2A), which was hypersensitive to fluconazole and cyclohexmide. A similar phenotype was observed when relative fluconazole (Fig. 2B) and cyclohexmide (Fig. 2 C) resistance of A1352M mutant, *BPDR5* and *pdr5Δ* mutant was determined in liquid media. Taken together, these results demonstrate that one single amino acid substitution of alanine 1352 with methionine is sufficient to impair Pdr5p function. In addition, the fact that all four single mutations separated from *PDR5-S2B* (S1360T, M1365I, S1368C and C1370S) could functionally complement the *pdr5*Δ mutant suggests the existence of synergistic effects among these mutations (Fig. 2A).

**Table 1:** Strains and plasmids.

Strains or plasmids	Relevant characteristics	Reference
Strains		
*S. cerevisiae* BY4741	*MATa his3 leu2 met15 ura3*	Yeast Knock-out (YKO) deletion collection
*S. cerevisiae* YJM789	*MATα hoΔ::hisG gal2 lys2*	[35]
*S. cerevisiae* G1	*MATa his3 leu2 met15 ura3 pdr5::loxP*	This study
*E. coli* DH5a	*SupE44, ΔlacU169 (φ80 lacZΔM15), hsdR17, recA1, endA1, gyrA96, thi-1, relA1, Nal^r^*	
Plasmids		
pUG6	*loxP-kanMX6-loxP*	EUROSCARF
pSH47	*URA3*, *Cre*	EUROSCARF
YEplac195	*URA3*	
pTA2		TOYOBO
YEplac195-*BPDR5*	*URA3*, expressing BY4741 *PDR5*	This study
YEplac195-*YPDR5*	*URA3*, expressing YJM789 *PDR5*	This study
*PDR5*-1		This study
*PDR5*-2		This study
*PDR5*-S1		This study
*PDR5*-S2		This study
*PDR5*-S2A		This study
*PDR5*-S2B		This study
*PDR5*-S2C		This study
YEplac195-*PDR5*-V1338I		This study
YEplac195-*PDR5*-L1344F		This study
YEplac195-*PDR5*-V1345A		This study
YEplac195-*PDR5*-N1349I		This study
YEplac195-*PDR5*-A1352M		This study
YEplac195-*PDR5*-S1360T		This study
YEplac195-*PDR5*-M1365I		This study
YEplac195-*PDR5*-S1368C		This study
YEplac195-*PDR5*-C1370S		This study
YEplac195-*PDR5*-A1352G		This study
YEplac195-*PDR5*-A1352V		This study
YEplac195-*PDR5*-A1352P		This study
YEplac195-*PDR5*-A1352S		This study
YEplac195-*PDR5*-A1352L		This study

### A1352M mutant strain is transport-deficient

**Figure 3:**
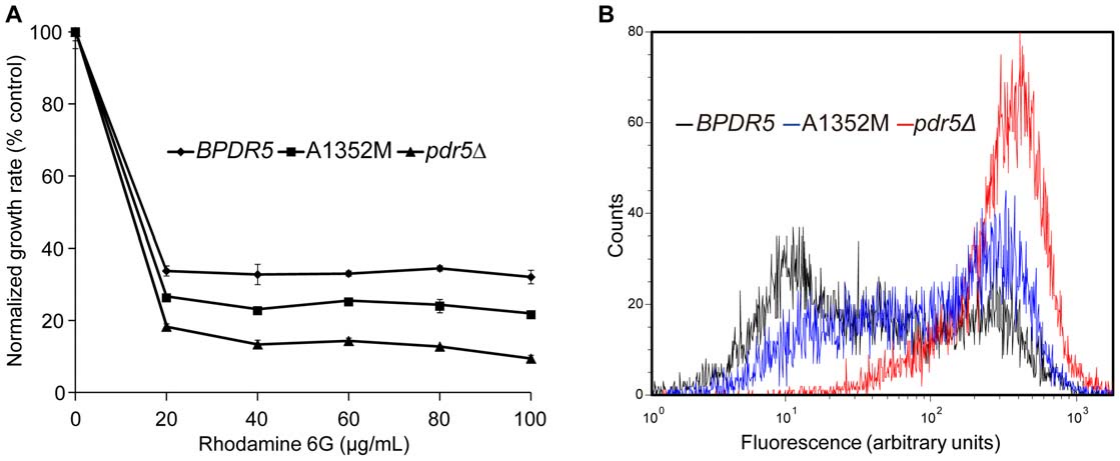
Pdr5p-mediated efflux of rhodamine 6G in *BPDR5* and mutant cells. (A) Rhodamine 6 G resistance of *BPDR5* (□), *pdr5Δ* (▴) and A1352 (▪) mutants were compared in liquid culture as described in materials and methods. Growth in the absence of R6G was served as control. Growth at each concentration of R6G is shown as (% control). The mean values of three independent experiments are plotted and the error bars represent the S.D. (B) The transport capabilities of *BPDR5*, *pdr5Δ* and A1352M were compared with flow cytometry. The histogram derived from the CellQuest software depicts fluorescence intensities for *BPDR5* (black), *pdr5Δ* (red) and A1352M (blue).

Furthermore, we analyzed the capability of A1352M mutant to transport rhodamine 6G (R6G) *in vivo*. Null mutants of *pdr5* were known to cause increased accumulation of R6G. As shown in Fig. 3A, A1352M mutant was more sensitive to R6G that *BPDR5*, the flow cytometry results (Fig. 3B) show that intracellular accumulation of R6G was much higher in A1352M mutant than that in cells expressing *BPDR5*. It demonstrates that transport capabilities of A1352M mutant are compromised. As a result, the observation that A1352M mutant conferred lower fluconazole and cycloheximide resistance than *BPDR5* resulted from transport deficiency of Pdr5p.

### Mutational analysis of A1352 in Pdr5p

**Figure 4:**
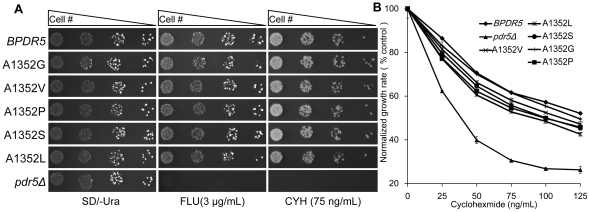
Drug resistance profiles of *pdr5Δ S. cerevisiae* cells expressing wild type and A1352 mutant variants of *PDR5* on agar plates and in liquid culture. (A) Drug sensitivity of A1352 mutant variants determined by spot assay. Serial dilutions of *BPDR5* cells, *pdr5Δ* and A1352 mutant variants were spotted on SD/-Ura agar plates in the absence (left panel) and the presence (right panel) of 75 ng/mL cycloheximide, respectively. The plates were incubated at 30°C for 72 hours. (B) Quantitative analysis of drug resistance mediated by Pdr5p mutants. Cells carrying empty plasmid or overexpressing *BPDR5*, A1352 mutant variants as indicated, were subjected to analysis in liquid culture as described in materials and methods. OD600 values at 12 h were analyzed. Growth in drug free cultures of each strain was served as control. Normalized growth rates are shown as the means ± standard deviations (error bars) for three independent experiments at each concentration of cycloheximide.

We further substituted the alanine residue in position 1352 with glycine (A1352G), serine (A1352S), proline (A1352P), valine (A1352V), and leucine (A1352L). The consequence of single residue changes in position 1352 to modulate Pdr5p mediated cycloheximide resistance was examined by spot assay after transformation of G1 with the corresponding overexpression plasmids. In order to compare the growth patterns of the mutants in different concentrations of cycloheximide, drug sensitivity analysis based on quantitative measurements of optical densities of liquid cell cultures was performed simultaneously as described in the method. As shown in Fig. 4, the capacities of A1352 mutants in conferring cycloheximide resistance were all slightly lower than that of *BPDR5*. The discrepancies of drug resistance for distinct mutations at position 1352 demonstrate that the size of residue at this position can affect the Pdr5p-mediated drug export.

### Mutations in A1352 elicit alteration in Pdr5p function

**Figure 5:**
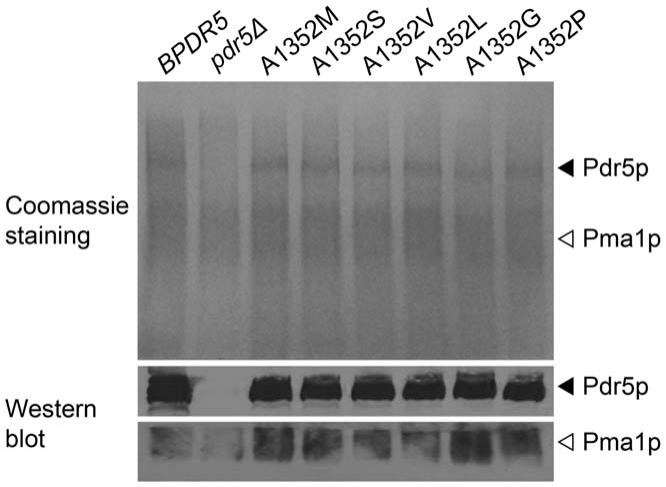
Membrane localization of Pdr5p and its mutant variants. Isolated plasma membranes (15 µg/lane) were electrophoresed on an SDS–8% polyacrylamide gel and visualized with Coomassie blue (top panel). Pdr5p (middle panel) and Pma1p (bottom panel) were immunodetected with specific antibodies as described in the materials and methods section. Filled arrowheads indicate Pdr5p and open arrowheads indicate Pma1p. Coomassie blue stained SDS-PAGE gel (upper panel) and western blot (lower panel) of the same strains are shown.

To ascertain that the observed differences in cycloheximide resistance are due to alterations in Pdr5p function rather than poor Pdr5p expression level or mislocalization of Pdr5p variants, plasma membranes from A1352 mutants were prepared and analyzed by SDS-PAGE. The presence of a specific band corresponded to Pdr5p in membranes from mutant variants of *PDR5* but was absent in membranes from *pdr5Δ* indicates that BY4741 Pdr5p and its A1352 mutant proteins are properly expressed and localized to plasma membrane with comparable levels (Fig. 5). This was further confirmed by immunoblotting with antibodies against Pdr5p (Fig. 5).

### Pdr5p-specific ATPase activity is unaffected by mutations in A1352

**Figure 6:**
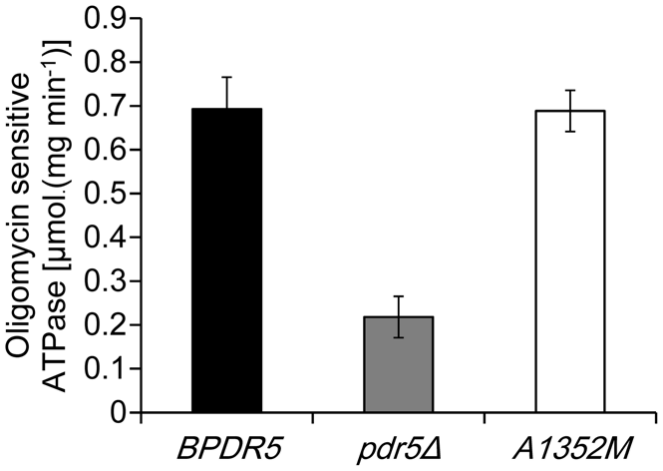
The effect of A1352M mutation on the ATPase activity of Pdr5p. Plasma membrane preparations (0.25 µg/well) from *BPDR5* (dark), *pdr5Δ* (gray) and A1352M (white) were incubated in 25 µL reaction buffer containing 300 mM Mes-Tris (pH 7.4), 4 mM ATP, 5 mM MgCl_2_, 0.2 mMammonium molybdate, 50 mM KNO_3_, and 10 mM NaN_3_ at 30°C for 20 min. The oligomycin-sensitive activity was determined as the difference in the ATPase activity in the presence or absence of 20 µg/mL oligomycin. The figure shows the means of three independent experiments, and the error bars represent standard error.

Energy released from ATP hydrolysis drives the substrate transport by Pdr5p. In order to examine whether the observed changes in cycloheximide resistance was accompanied by alterations in Pdr5p-specific ATPase activities, plasma membranes isolated from mutants carrying Pdr5p variants were used to determine their ATPase activities. It is apparent from Fig. 6 that, compared to plasma membranes from the cells expressing *BPDR5*, there was no significant difference in plasma membrane ATPase activity of cells expressing mutant *PDR5*, the plasma membranes from *pdr5* null mutant exhibit considerably reduced ATPase activity. Therefore, mutations at A1352 do not seem to interfere with Pdr5p-specific ATPase activity.

## Discussion

*S. cerevisiae* Pdr5p belongs to the ABC super-family of primary transporters which directly couples ATP hydrolysis to substrate transport. It not only expels azoles and its derivatives but also extrudes a variety of structurally unrelated compounds [1], [26]. To understand the structure-function relationships of Pdr5p, we took advantage of the highly polymorphic Pdr5p from clinical strain YJM789 that have lost its ability to export azoles. By exchanging segments of BY4741 *PDR5* with their counterparts from YJM789 *PDR5*, we found that A1352M substitution could cause considerable reduction in Pdr5p mediated drug resistance.

As shown in Fig. 1B, Pdr5p shares a common architecture with other ABC transporters, which contains two TMDs and each of the two is comprised of six membrane-spanning helices connected by three extracellular loops (ECLs) and two intracellular loops (ICLs). Recent molecular modeling study on Pdr5p [11] shows that A1352 (The purple sphere indicated by arrow in Fig. 1B) is located in the ICL4 which connects TMH 10 and TMH 11 in TMD2. It is widely accepted that interactions between the membrane-bound domains and cytosolic domains of ABC transporters are critical for their transport capabilities. The high-resolution structure of *Staphylococcus aureus* Sav1866 suggests that the NBDs interact with the TMDs via ICLs [27]. Analysis of P-glycoprotein by chemical cross-linking suggests that ICL4 within TMD2 forms an interface with NBD1 [28]. Studies of CFTR (Cystic Fibrosis Transmembrane Regulator) mutants demonstrate a tertiary interaction between the surface of NBD1 and ICL4 in TMD2 [29]. ICL4-NBD1 interaction in Yor1p (another ABC transporter in *S. cerevisea*) has been defined by chemical cross-linking experiments. [30]. All above studies supported an interface with a trans arrangement in which ICL4 within TMD2 interacts with the NBD of the opposite half of the molecule.

Despite their distinct molecular architecture, it could be inferred from the predicted topological models that ICL4 in Pdr5p corresponds to ICL4 in above mentioned ABC transporters. It is possible that the ICL4 of Pdr5p is involved in interdomain cross-talk. Reduction of drug resistance in A1352M mutant may attributable to the alterations of ICL4 that uncouple interdomain cross-talk. However, substitution of A1352 with more rigid amino acid residue proline does not show significant reduction in the drug resistance and the mutant possess the same level of ATPase activity as *BPDR5* (data not shown). Genetic and biochemical studies from Pdr5p strongly suggest that the Pdr5p signaling interface is in the cis rather than the trans configuration, interactions between the TMH2 and NBD1 occur via the ICL1 [13], [14].Therefore, it is unlikely that the reduced resistance to drug substrates in the A1352M mutant was a consequence of uncoupling of interdomain cross-talk.

The reported crystal structures of Sav1866 [27] and mouse P-glycoprotein [31] suggest that the transmembrane helices in TMDs form a substrate-binding pocket. Modeling of Pdr5p shows that Ala1352 is pointing toward the predicted substrate-binding pocket [11]. It is more likely that the transport-deficiency of A1352M mutant is caused by the increased length of residue which results in affecting accessibility of binding pocket to intracellular substrates. We also increased the residue size at position 1352 by substituting the Ala with Gly (A1352G), Ser (A1352S), Val (A1352V) and Leu (A1352L). Western blot result showed that the expression levels of all above A1352 mutants were similar to that of *BPDR5*. The drug resistance assays also support that substitutions of Ala residue with larger residues tend to have more severe impact on Pdr5p, resulting in increased sensitivity to cycloheximide.

Previous work shows that A1352 and the negatively charged E1353 in ICL4 are very conserved by aligning *PDR5* with other fungal ABC transporters, and E1353 is predicted to located at the edge of substrate-binding pocket [11].When positively charged Lys (K) and uncharged Gln (Q) were used to substitute the similar-size E1353, only E1353K mutant shows significant decrease in drug efflux. (data not shown). This implies that the E1353 may play a role in neutralizing positively charged substrates such as rhodamine 6G. It requires further investigation to understand whether the importance comes from its role in substrate recognition.

Interestingly, the A1352 was identified to be under positive selection by branch-site model (*P* ω>1, posterior probability of NEB was 0.98, and BEB was 0.93). Although this significance is not extremely high, the fast evolution of A1352 indicates that this amino acid is responsible for the changing of Pdr5p function in drug transport.

In summary, mutation of A1352 in ICL4 could disturb Pdr5p mediated drug resistance pattern without affecting its ATPase activity, expression level or protein localization. It hints that the ICL4 of Pdr5p may play an important role in substrates recognition and export. While, there is no report about ICL4 function in Pdr5p, our observation of the functional importance of A1352 might encourage further studies on this region.

## Materials and Methods

### Strains, media and growth conditions

*S. cerevisiae* BY4741 (*MAT a, his3Δ, leu2Δ, met15Δ, ura3Δ*) and YJM789 (*MAT α, hoΔ::hisG, gal2Δ, lys2Δ*) were used for *PDR5* cloning. *pdr5* null mutant G1 (*MAT a, his3Δ, leu2Δ, met15Δ, ura3Δ, pdr5Δ::loxP*) was constructed as previously described [32]. *Escherichia coli* strain DH5α was used for plasmid manipulation.

*E. coli* cells were grown in LB medium. Yeast strains were grown on standard rich YPD medium. SD uracil-deficient medium was used to select *ura3* auxotrophic derivatives of transformants. Solid media were supplemented with 2% agar.

### Chemicals

Stock solutions of fluconazole (FLC) and Rhodamine 6G (R6G) were prepared in 20% dimethyl sulfoxide at final concentrations of 5 mg/mL and 10 mM. Cycloheximide (CYH) solution (1 mg/mL) was purchased from Sigma-Aldrich.

### Plasmid construction and transformation

Construction of plasmids YEplac195-*BPDR5*, YEplac195-*YPDR5*, YEplac195-*PDR5*-1 and YEplac195-*PDR5*-2 has been described previously [22]. An 1851 bp segment was amplified by PCR from YEplac195-*YPDR5* with forward primer #01 and reverse primer #02. The PCR product was digested with *BstP* I and *Mlu* I and ligated to YEplac195-*BPDR5* cut accordingly producing *PDR5-S1*. *PDR5-S2* was similarly generated using forward primer #03, reverse primer #04 and *Mlu* I – *Sph* I as restriction enzymes. The PCR product of primer pair #03 and #04 was also ligated into the pTA2 vector to obtain pTA-S2 for further modification.

For substituting blocks S2C in YEplac195-*BPDR5* by its counterpart from YEplac195-*YPDR5*, YEplac195-*BPDR5* was cut with restriction enzymes *Pst* I - *Bsp1407* I, the resulted restriction fragment of 209 bp was recovered and used to replace its counterpart sequence in pTA-S2, yielding intermediate plasmids pTA-S2C; pTA-S2C was then digested with single restriction site enzymes *Mlu* I and *Sph* I and ligated to YEplac195-*BPDR5* cut accordingly, creating *PDR5-S2C*.

*PDR5-S2A* and *PDR5-S2B* were constructed as follows. Inverse PCR amplification was performed using PAGE-grade PCR primers #05 and #06 which containing the desired non-synonymous base pair substitutions indicated by uppercase letters and pTA-S2C plasmid DNA isolated from *E. coli* DH5α as template. After *Dpn* I digestion of the methylated template plasmid, self-ligation of PCR product was conducted in a reaction mixture which includes both T4 Polynucleotide Kinase (TOYOBO) and T4 DNA ligase (Fermentas). The resulted plasmid was labeled pTA-S2A. The same procedure was applied to construct pTA-S2B using primer pair #07 and #08. Substitutions of nucleotides introduced by primers were confirmed by DNA sequencing. *Mlu* I – *Sph* I fragments from pTA-S2A and pTA-S2B were used to replace the homologous fragments in YEplac195-*BPDR5* generating *PDR5-S2B* and *PDR5-S2A*, respectively. The sequences of primers used for plasmid construction are listed in Table 2.

**Table 2:** Primers used in construction of *PDR5* chimeras.

Primer	Sequence(5′-3′)
#01	CATTAATCCATTGGCTTACTT
#02	aactacgcgtTAGAGTAAAACCCG
#03	atatacgcgtCTGCAGCTGGACAG
#04	acatgcatgcACCGATGAGATAACCTAGAAAT
#05	tctttta**AC**caagtt**GCA**gaaagtgctgcaaacttag
#06	aat**GA**caa**G**tagacccattgaaccaa**C**gtaaacgtag
#07	tctttat**C**tttct**G**tggtgttatgaccacacccag
#08	**C**atggtgaacaataaag**A**tgctaagtttgcagcac

Bold: modified nucleotides.

Underlined: modified or newly generated endonuclease restriction sites.

Yeast transformations were performed by LiAc/SS carrier DNA/PEG high efficiency transformation protocol [33].

### Site-directed mutagenesis of *PDR5*

The *Mlu* I – *Sph* I DNA fragment from YEplac195-*BPDR5* was used to replace the homologous fragment in pTA-S2 creating pTA-mu. pTA-mu was then served as the template for site-directed mutagenesis to create *PDR5* mutants. Site directed mutagenesis was performed using the Quick Change kit (Stratagene). Mutations were introduced into YEplac195-*BPDR5* from pTA-mu derivatives bearing desired nucleotide substitutions by exchanging the *Mlu* I – *Sph* I fragments in plasmids.

All PCR amplifications were performed with the KOD-Plus system (TOYOBO). After every cloning, the entire chimeric construct was sequenced by using the Big Dye Terminator Cycle sequencing kit and an ABI 3730xl DNA analyzer to ensure that the construct remained in-frame and no unwanted mutations had been introduced.

### Drug sensitivity analysis

Each transformed yeast strain to be tested was grown in SD/-Ura medium to late exponential phase. Then cells were washed and subsequently resuspended in sterile water to an OD600 of 0.1. For spot assay, aliquots (4 µL) of 5-fold serial dilutions were spotted onto SD/-Ura plates lacking or containing drugs to be tested. Plates were incubated at 30°C for 72 hours. For quantitative measurements of optical densities of liquid cell cultures, cultures were treated with different concentrations of cycloheximide. 150 µL normalized cultures were seeded onto NUNC 96 well optical bottom plates (in triplicate) and covered with adhesive plate sealers in Power Wave XS microplate spectrophotometer (BioTek) with constant shaking at 30°C. OD600s for each well were recorded using Gen5 software every 15 minutes for 24 hours.

### Preparation of purified membrane vesicles and immunoblots

Plasma membranes were prepared as described previously [15]. The protein concentration was determined by Bradford assay. Equivalent amounts of protein were separated by SDS-PAGE with 8% gel. Immunoblotting was done using standard methods. Pdr5p specific goat polyclonal antibody yN-18 and Pma1 specific antibody yN-20 were from Santa Cruz Biotechnology. HRP labeled donkey anti-goat polyclonal antibody was from Beyotime Co. Proteins were visualized using the ECL chemiluminescence detection system (BeyoECL Plus).

### ATPase activity assays

Pdr5p-associated OM-sensitive ATPase activity of the isolated plasma membrane fractions was measured as described previously [15]. Absorbance data were collected using a Power Wave XS microplate spectrophotometer equipped with Gen5 software (BioTek).

### Rhodamine 6G transport assay

Transport of rhodamine 6G was determined as described previously [13]. The data were analyzed with a CellQuest program. Fluorescence was expressed in arbitrary units.

### Detecting positive selection

10 *PDR5* gene sequences of different *S. cerevisiae* strains were selected from website (http://www.sanger.ac.uk/research/projects/genomeinformatics/sgrp.html). Pdr5p sequences were aligned using ClustalW with the default parameter settings, and were then reversely translated into nucleotide sequences using MEGA4 [34]. The evolutionary rate of amino acid sites was detected by using the branch-site model of positive selection, as implemented in codeml program in PAML package [27].
